# Comparison between immediately and next day post RF ablation MRI in patients with atrial fibrillation

**DOI:** 10.1186/1532-429X-14-S1-P200

**Published:** 2012-02-01

**Authors:** Eugene Kholmovski, Sathya Vijayakumar, Christopher McGann, Nassir F Marrouche

**Affiliations:** 1 UCAIR, Department of Radiology, University of Utah, Salt Lake City, UT, USA; 2 CARMA Center, University of Utah, Salt Lake City, UT, USA

## Background

Atrial fibrillation (AF) is the most common cardiac arrhythmia affecting more than 5 million people in North America and Europe. Radio-frequency ablation (RFA) is effective in symptomatic, drug refractory AF. Reported success rates of the procedure vary significantly with AF recurrences ranging from 25-40%. The transmural extent of left atrial (LA) wall injury is difficult to assess with conventional electro-physiological (EP) measurements. Late gadolinium enhancement (LGE) and double inversion recovery T2-weighted TSE (DIR-T2w-TSE) and HASTE have been proposed to evaluate acute LA wall injury. In this study, we examine the evolution of acute (< 30 hours after RFA) atrial lesions in LGE and DIR-T2w-TSE MRI with time after the procedure. The main goals of this project are to study changes in acute lesion appearance with time after RF ablation and to determine the time interval after ablation when MRI achieves the best visibility of acute LA lesions.

## Methods

From January 2010 to September 2011, 224 AF patients who underwent pulmonary vein isolation and debulking of the septal and posterior walls under EAM guidance (CARTO, Biosense Webster) were imaged on a 3 Tesla MR scanner (Verio, Siemens Healthcare). Typical time interval between the conclusion of the ablation procedure and patient in the scanner was less than an hour. MRI studies were performed to rule out procedure complications and assess the extent of injury to LA wall. The study protocol included DIR-T2w-TSE, contrast enhanced MR angiography (Multihance, 0.1 mmol/kg), and 3D LGE scans. Ten patients underwent an additional MRI study on the next day after ablation (22.0±2.6 hours) to follow-up on significant enhancement of the anterior wall of the esophagus in vicinity of LA wall, detected by immediately post-ablation LGE-MRI. Contrast-to-noise ratio (CNR) between LA lesion and blood (CNR_LB_) and between LA lesion and normal myocardium (CNR_LM_) were evaluated for these patients using immediately and next day post-ablation DIR-T2w-TSE and LGE images. Signal intensity for LA lesion/blood/normal myocardium was measured in LA posterior wall/LA cavity/LV wall, correspondingly. Standard deviation of noise was assumed to be equal to standard deviation of blood signal in the LA cavity.

## Results

**Figure 1:**
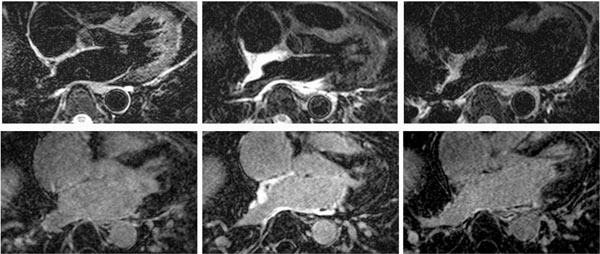
DIR-T2w-TSE (top row) and LGE (low row) images of AF patient. Left column - pre-ablation, middle column - immediately post-ablation, right column - next day post-ablation. Notice high contrast between ablated regions of LA wall (posterior wall, septum, ostia of right posterior PV) and the other tissues in immediately post-ablation DIR-T2w-TSE and LGE images.

Quantitative analysis (Table 1) and visual inspection (Fig.1) of the DIR-T2w-TSE and LGE images indicate that contrast between ablated regions of LA wall and the other tissues is significantly higher in immediately post-ablation images than in the next day post-ablation images.

**Table 1:** CNR_LM_ and CNR_LB_ in immediately and next day post-ablation DIR-T2w-TSE and LGE-MRI

	DIR-T2w-TSE			LGE-MRI		
	
	Immed. Post	Next Day	p-value	Immed. Post	Next Day	p-value
CNR_LM_	17.53±3.04	8.90±4.35	0.001	16.47±2.17	11.92±2.62	0.0005
CNR_LB_	23.24±4.96	13.76±5.38	0.005	9.17±2.36	3.75±2.52	0.0001

## Conclusions

The presented results indicate a fast physiological response of LA wall to injury. Edema is noticeably reduced 24 hours after RF ablation. Kinetics of contrast agent in injured regions changes considerably during the first 24 hours after RF ablation. Our results demonstrate that MRI study should be performed as soon as possible after conclusion of the RFA procedure to achieve high contrast between ablated LA wall and the other tissues.

## Funding

Ben B. and Iris M. Margolis Foundation.

